# Development and evaluation of ActSeq: A targeted next-generation sequencing panel for clinical oncology use

**DOI:** 10.1371/journal.pone.0266914

**Published:** 2022-04-21

**Authors:** Zonggao Shi, Jacqueline Lopez, William Kalliney, Bobbie Sutton, Joyce Simpson, Kevin Maggert, Sheng Liu, Jun Wan, M. Sharon Stack

**Affiliations:** 1 Harper Cancer Research Institute and Department of Chemistry and Biochemistry, University of Notre Dame, Notre Dame, Indiana, United States of America; 2 Genomics and Bioinformatics Core Facility, University of Notre Dame, Notre Dame, Indiana, United States of America; 3 South Bend Medical Foundation, South Bend, Indiana, United States of America; 4 Collaborative Core for Cancer Bioinformatics and Department of Medical and Molecular Genetics, Indiana University School of Medicine, Indianapolis, Indiana, United States of America; CNR, ITALY

## Abstract

**Purpose:**

The demand for high-throughput genetic profiling of somatic mutations in cancer tissues is growing. We sought to establish a targeted next generation sequencing (NGS) panel test for clinical oncology practice.

**Methods:**

Customized probes were designed to capture exonic regions of 141 genes selected for the panel, which was aimed for the detection of clinically actionable genetic variations in cancer, including *KRAS*, *NRAS*, *BRAF*, *ALK*, *ROS1*, *KIT* and *EGFR*. The size of entire targeted regions is 0.8 Mb. Library preparation used NEBNext Ultra II FS kit coupled with target enrichment. Paired-end sequencing was run on Illumina NextSeq 500 at a read length of 150 nt. A bioinformatics workflow focusing on single nucleotide variant and short insertions and deletions (SNV/indel) discovery was established using open source, in-house and commercial software tools. Standard reference DNA samples were used in testing the sensitivity and precision and limit of detection in variant calling.

**Results:**

The general performance of the panel was observed in pilot runs. Average total reads per sample ranged from 30 million to 48 million, 73% ~82% unique reads. All runs had more than 99% average mapping rate. Mean target coverage ranged from 727x to 879x. Depth of coverage at 50x or more reached 87% of targeted region and 60% of targeted region received 500x or more coverage depth. Using OncoSpan HD827 DNA, which bears 144 variants (SNV/indel) from 80 genes that are within the targeted region on the panel, our somatic variant calling pipeline reached 97% sensitivity and 100% precision respectively, with near 48 million reads. High concordance with orthogonal approaches in variant detection was further verified with 7 cancer cell lines and 45 clinical specimens.

**Conclusion:**

We developed a NGS panel with a focus on clinically actionable gene mutations and validated the performance in library construction, sequencing and variant calling. High concordance with reference materials and orthogonal mutation detection was observed.

## Introduction

Next generation sequencing (NGS) technologies are transforming the practice of many areas in clinical molecular medicine. Identifying key oncogenic drivers and actionable genetic alterations in a high throughput manner has been increasingly adopted in current clinical oncology practice, benefiting the diagnostic, prognostic and therapeutic needs of many cancer patients. For example, National Comprehensive Cancer Network (NCCN) guidelines (version 2.2018) recommend *KRAS*, *NRAS* and *BRAF* mutation detection in metastatic colorectal carcinoma(CRC) for the well-established role in predicting therapeutic response [1]. CRC with mutations in exons 2–4 of *KRAS* or *NRAS* genes are not eligible for *EGFR* antibody therapies [2]. The College of American Pathologists and the Association of Molecular Pathology guidelines recommend *EGFR*, *ALK* and *ROS1* gene tests as mandatory in non-small cell lung carcinoma(NSCLC) [3].

The pressing clinical need to characterize the genetic profile of individual tumors has led to a surge in genome-wide tests [4,5], and recently clinical whole genome sequencing and whole exome sequencing have been in use in some academic tertiary cancer centers [6]. However, for cost-effectiveness and operational feasibility, targeted panel-based NGS assays, such as those represented by FoundationOne [7] and MSK-IMPACT [8] are still the mainstream applications in oncology practice.

Clinical NGS panel tests vary considerably in design and implementation across different laboratories. Some chose to validate pre-designed panels from vendors and others customized their own panels. To newcomers in the field, it remains a challenge to establish a targeted panel test from scratch since a significant investment of operational and bioinformatics infrastructure is required. But the benefits are also obvious, as newly identified actionable target regions can be readily added to the panel to meet ever-evolving clinical needs. Considering the fact that approximately 80% of cancer patients in the USA are treated locally at community hospitals [9], where resources are not readily available with regard to clinical NGS panel testing and the related training or expertise to interpret these results, it is meaningful to experiment and expand the capability of customizing a clinical NGS panel test.

Herein we describe our experience in developing a NGS-based comprehensive panel that includes actionable small variants (SNVs and indels) in common cancer types, examining and refining its performance for a potential implementation in a community pathology laboratory that would therefore benefit the practice of oncology in the region. We developed a hybridization capture-based NGS panel test for cancer mutation, designated ActSeq, with the focus on SNV/indel variants of 141 cancer genes in solid tumors and leukemia, and evaluated the detection performance in FFPE specimens using the NextSeq 500 (Illumina, CA) platform.

## Materials and methods

### ActSeq panel design

For best clinical utility, the selection of genes was primarily based on those with FDA approved targeted therapies [10], OncoKB actionable genes [11], the molecular test menu of our local clinical pathology laboratory and recommendations from local pathologists and oncologists. The panel was aimed to provide coverage of a total of 141 cancer-related genes (S1 Table: Gene list). All the current actionable genes, which bear mutations with known targeted therapy or otherwise guide clinical therapy, are included. The size of entire targeted space is about 0.8Mb. Probes for target capture were custom-designed and manufactured with MYBaits technology from Arbor Bioscience (Ann Arbor, MI). MYBait probe/bait is 80-mer in length and allows more than 5% difference between target and probes/baits. The ActSeq panel used 19240 probes (illustrated in S1 Fig and sequences provided in S2 Table) at 2x tiling for targeted regions on 141 genes (3044 exons), with a total bait territory of about 1.0Mb. Considering the targeted space, bait design efficiency is 0.87.

### Tissue, cell and DNA samples

A standardized reference cell line NA12878 from Genome-In-A-Bottle (GIAB) project was purchased from Coriell Cell Repositories (Camden, NJ, USA). The truth set for variants in the NA12878 cell line was obtained via the website precision.FDA.gov. OncoSpan DNA HD827 was purchased from Horizon Discovery (Cambridge, UK). Seven ovarian and breast cancer cell lines, SKOV3, OVCAR5, OVCAR8, MDAMB231, MDAMB468, HCC38 and HCC1806, were all originally obtained from ATCC (Manassas, VA) and propagated in our research lab. Information on the genetic variants (SNV/indels) within these cell lines was obtained via the website of the Cancer Cell Line Encyclopedia (CCLE) project and manually curated (https://sites.broadinstitute.org/ccle/).

All human cancer tissue samples were retrospectively obtained from South Bend Medical Foundation, South Bend, IN with a waiver of informed consent approved by the Institutional Review Board (IRB exempt protocol #17-11-4231). All cases were diagnostically confirmed by consultant pathologists. DNA preparation from fresh tissue or cells was performed as previously described [12]. For clinical formalin-fixed paraffin-embedded (FFPE) tumor samples, the QIAmp DNA FFFE Tissue kit was used. Selected tumor tissue blocks (tissue > = 0.5 cm in size, at least 20% tumor cells in circled area) were sectioned 8 μm thick (5 sections per case). With H&E tissue slide where regions with tumor were marked by pathologists as a reference, non-tumor was removed by dissection. Sections were processed as follows: remove paraffin in xylene; lyse under denaturing conditions with proteinase K, reverse formalin crosslinking by incubation at 90°C; bind DNA to the membrane and wash away contaminants, and finally elute the DNA with Tris-EDTA buffer.

### Mutation detection with OncoFOCUS MassArray panel

Agena OncoFOCUS Panel v3.0 is based on the MassArray System (Agena Bioscience, San Diego, CA, USA) for the detection and quantification of 230 driver mutations in BRAF, EGFR, KRAS, NRAS, and KIT. It employs matrix-assisted laser desorption/ionization time-of-flight mass spectrometry for amplicon detection and differentiation [13]. Primers were pre-designed and provided by the manufacturer for PCR (polymerase chain reaction) amplification of regions with specific mutations. PCR reactions contained Taq DNA polymerase, genomic DNA (5~10 ng), PCR primers, and dNTP. Following PCR (45 cycles), the remaining dNTPs were removed by the addition of shrimp alkaline phosphatase (SAP), after which the plates were incubated at 37°C for 40 min. Following the PCR reaction, SAP addition, and extension reaction, the samples were desalted by resin treatment for 15 min, then spotted onto SpectroCHIP^®^ Arrays and analyzed by mass spectrometry. SpectroTYPER v4.0 software (Agena Bioscience, San Diego, CA) was used for the ultimate interpretation of the results.

### Library preparation and target capture

NEBNext Ultra II FS DNA library prep kit (NEB Lab, MA, USA) was used for NGS library preparation. All starting DNA samples were quantified with Qubit 2.0 Flurometer (Thermo Fisher, Waltham, MA) and starting DNA amount was 100ng for a standard test (range of 20~200ng). Fragmentation of DNA to 200–450 bp size was carried out at 37 °C for 10 min with 2 μl NEBNext Ultra II FS enzyme mix, 7 μl reaction buffer in a total volume of 35 μl with DNA, followed by end preparation (30 minutes at 65 °C), then proceeded to adaptor ligation, which was carried out at 20 °C for 15 minutes after the addition of 2.5 μl NEBNext adapter for Illumina, 1.0 μl NEBNext Ultra II Ligation enhancer and 30.0 μl NEBNext Ultra II ligation master mix. For all library preparations starting with 100ng or more DNA, size selection with 2 rounds of NEBNext Sample Purification Beads was performed. All adapter-ligated DNA preps were PCR amplified (6 cycles) for enrichment and cleaned up with one round of Sample Purification Beads. Finally, libraries were checked with Bioanalyzer (Agilent, Santa Clara, CA) for size quality control and quantified with Qubit 2.0 Flurometer.

To enrich the target DNA specific to the regions that the ActSeq panel was designed to detect, customized biotinylated RNA baits MYBaits (Arbor Bioscience, Ann Arbor, MI) selection was applied. Briefly, every four properly prepared and indexed DNA libraries were pooled and concentrated with AMPure XP beads (Beckman Coulter, Indianapolis, IN) as one sample. For each capture reaction, per user manual version 3.01, 5.0μl Blockers mix and 7μl of pooled library input were used for a 30 μl total reaction volume. It was then heat-denatured in the presence of adapter-specific blocking oligonucleotides, which were used to bind to library adapters before biotinylated RNA baits were introduced for hybridization at 65 °C for 24 hr with lid at 105^°^C. Streptavidin-coated magnetic beads (30μl for each capture reaction, Dynabeads^®^ MyOne^™^ Streptavidin C1 magnetic beads from Invitrogen, #650–01) were used to pull out bait-target hybrids. Beads were stringently washed 4 times and finally the captured DNA library was eluted from beads, followed by PCR amplification (11 cycles), then cleaned up with AMPsure XP beads. The final library pool was quantified by KAPA library qPCR quantification Kit (Roche Sequencing, Pleasanton, CA). All sequencing was run on Illumina NextSeq 500, with paired end reading length of 150 nt (NextSeq 500/550 Mid Output v2 Kit, 300 cycles).

### Bioinformatics pipelines and data analysis

NGS data processing was done on the high-performance computing cluster at the University of Notre Dame Center for Research Computing using a pipeline developed with open-source software tools. A schematic overview of the major steps is provided in S2 Fig. Raw reads in de-multiplexed FASTQ files were run by FastQC (v0.11.8), and then aggregated with MultiQC (v1.8) for manual read quality inspection. All read alignments were performed with BWA-MEM (v0.7.17) and human genome reference hg19 as provided by GATK bundle (https://gatk.broadinstitute.org/). SAMtools (1.9), GATK (v4.2.0.0, including Mutect2) and ANNOVAR (v20210123) were used in quality control metrics, deduplication, base quality recalibration, local realignment, variant calling and variant annotation. Variant calls were limited to predefined target regions and without paired normal control. The removal of germline polymorphism was achieved by filtering against population databases, including dbSNP(Common 151) and gnomAD(v2.1), and all variants with minor allele frequency >1% in the databases were excluded. VarSeq (v2.0.2) from Golden Helix, Inc. (Bozeman, MT) was used for variant filtration and annotation of clinical samples. In particular, variants that met any one of the following, VAF (variant allele frequency) <0.05, read depth < 50x or variant supporting reads <5 were removed. Manual review was used for variant validity confirmation. Integrative Genomics Viewer (IGV, v2.11.0) was used for manual data inspection and visualization. Raw data from all cell line samples are available from SRA (BioProject PRJNA803819) and the bioinformatics pipeline (snakemake v6.14.0) is available on GitHub (https://github.com/harpernd/actseq, release v1.0.0). Raw data from patient samples are not publicly sharable per IRB and dbGaP repository policy.

RTGtools vcfeval from Realtime Genomics (Hamilton, New Zealand) was used to evaluate VCF (Variant Call Format) outputs from variant calling in comparison with known truth set VCF file from the DNA sample supplier. Performance metrics include sensitivity, precision and F-score. For sensitivity estimation, based on the truth set, variants were classified as true positive (TP) or false negative (FN) if not detected. Sensitivity was expressed as TP/(TP+FN). Precision estimation was based on variants call true positive (TP) or false positive (FP) if not in truth set and it was calculated as TP/(TP+FP), this is also known as positive predictive value (PPV). F1 score was calculated as 2TP/(2TP+FP+FN), which is the harmonic mean of precision and sensitivity. For statistical significance of group comparisons, Welch’s *t*-test was used and significance level set at α<0.01.

## Results

### Characteristics of ActSeq next generation sequencing panel

**Fig 1:**
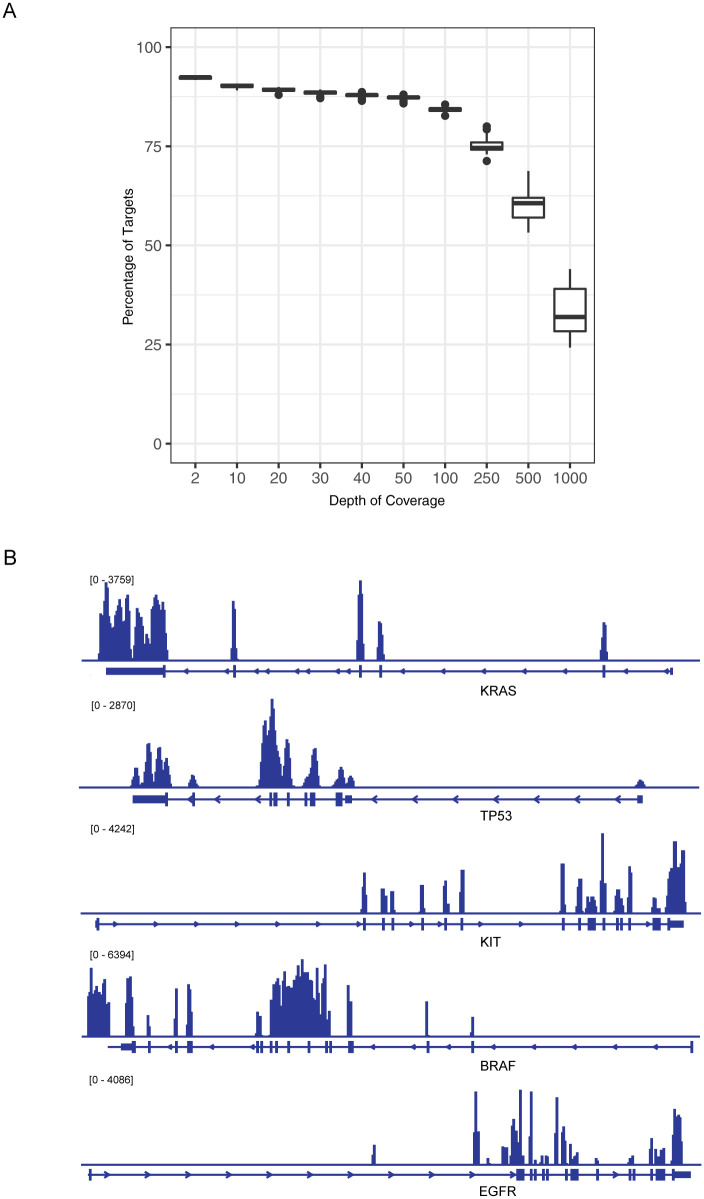
Coverage of ActSeq next-generation sequencing panel. A) Depth of coverage and corresponding percentage of target regions as observed in pilot runs. B) Coverage profile on selected cancer genes in HD827 data as extracted with IGV.

Sequencing reactions with the ActSeq panel were run in 10 batches, yielding a mean of 39 million reads per sample (range 23~62 million) with base quality above Phred score 30. In Table 1, basic characteristics were extracted from the first 3 batches of samples, 8 samples per run, and reflected the general performance of this panel design. Total reads per sample range from 30 million to 48 million, 73% ~82% of reads are unique (i. e. not duplicates), off-bait reads are 53% ~58%. All samples had more than 99% mapping rate. Less than 4% of targeted regions were not covered. The mean target coverage ranges from 727x to 879x. As shown in Fig 1A, a depth of coverage at 50x or more was reached for 87% of targeted regions and 60% of targeted regions received 500x or more coverage depth. The coverage on exonic regions of individual genes is illustrated in Fig 1B.

**Table 1:** Performance metrics of ActSeq panel.

	RUN01(n = 8)			RUN02(n = 8)			RUN03(n = 8)		
	mean	min	max	mean	min	max	mean	min	max
Total Reads	39M	35M	46M	48M	42M	54M	31M	28M	34M
Unique Reads (%)	73.19%	72.16%	74.88%	77.60%	73.06%	83.17%	82.35%	79.60%	84.08%
Mapping Rate (%)	99.39%	99.33%	99.45%	99.14%	98.71%	99.31%	99.41%	99.37%	99.45%
Mean Bait Coverage	1804.14	1543.18	2015.02	2108.85	1807.57	2562.26	1235.35	1098.47	1444.21
Off Bait Reads(%)	52.90%	51.16%	55.28%	57.69%	54.77%	65.56%	56.63%	52.32%	58.61%
Mean Target Coverage	878.96	799.62	977.00	757.46	671.91	879.27	727.35	661.43	798.51
Targeted Region Not Covered (%)	3.26%	2.96%	3.78%	3.01%	2.66%	3.29%	3.23%	2.69%	4.37%

**Fig 2:**
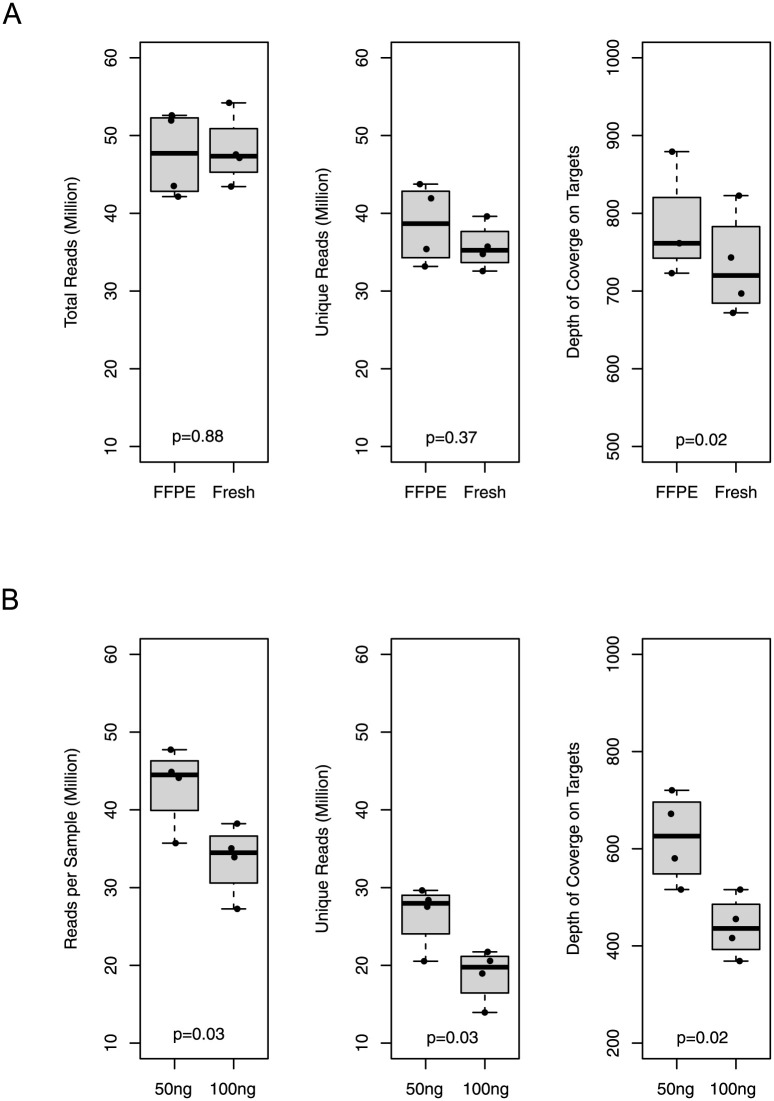
Sequencing performance of DNA source and input amount. A) Comparison of equal amount of input DNA from fresh-frozen tissue vs. from formalin-fixed paraffin-embedded (FFPE) materials. B) Comparison of 50ng without size selection vs 100ng with size selection input DNA in library preparation.

Due to the dominant use of FFPE material in pathology labs, we compared the metrics of library preparation and sequencing of DNA samples from fresh frozen material vs those from FFPE samples (Fig 2A). When using a starting DNA amount of 100ng and a bead-based size selection method, no major difference was discovered between the fresh group and FFPE group (4 samples in each group) in terms of total reads, unique reads, or depth of coverage on targeted regions.

Using DNA from FFPE materials, we compared the impact of different starting amount of DNA on panel performance. Based on the recommendation from NEBNext Ultra II FS DNA library prep kit manual, when the DNA amount is less than 100ng, standard library size selection was not applied, instead one round of the clean-up procedure was used. Interestingly, it was observed that using DNA samples from the same 4 patients, the group with 50ng of starting DNA (without size selection) exhibited a higher yield of reads, higher number of unique reads and higher depth of coverage on targets than those starting with 100ng DNA as show in Fig 2B (although not statistically significant). Even lower amounts of starting DNA amount are also feasible. The lowest amount of DNA we used was 13ng (Sample H21023), which still produced a total of 44 million reads, 15 million unique reads and reached a mean target coverage at 52x. The known variant in this sample was clearly identified by our ActSeq NGS panel test.

### Performance in variant detection with reference materials

The variant calling pipeline for the ActSeq panel was established following GATK best practice [14] and the workflow is illustrated in S2 Fig. Although the ActSeq panel is meant for somatic mutation detection, to gain familiarity with the variant calling and filtering process we purchased DNA from NA12878 cells. This sample is from a transformed B-Lymphocyte cell line that has been extensively tested with NGS methods and is now often used as reference sample to NGS variant calling workflows, a “genome-in-a-bottle” as promoted by National Institute of Standards and Technology (NIST) [15]. This sample was run through the ActSeq panel using a separate germline variant calling pipeline made with GATK HaplotypeCaller. High sensitivity, precision (also known as positive prediction value, PPV) and F1 score were obtained for NA12878 germline variant calls as shown in the first row of Table 2.

**Table 2:** Sensitivity and precision of ActSeq panel.

DNA	PercentageReads	True-pos	False-pos	False-neg	Sensitivity	Precision	F1-score
NA12878(54M reads)	100%	1355	22	33	98%	98%	98%
HD827(48M reads)	100%	139	0	5	97%	100%	98%
HD827	50%	139	0	5	97%	100%	98%
HD827	10%	121	0	23	84%	100%	91%
HD827	5%	101	0	43	70%	100%	82%
HD827	1%	48	0	96	33%	100%	50%

For somatic variant calling, we used OncoSpan HD827 DNA sample for performance evaluation. According to the supplier, OncoSpan is a well-characterized, cell line-derived reference standard containing 386 variants across 152 key cancer genes. Among them, 80 genes (144 variants) are within the targeted region on the ActSeq panel. Our MuTect2 based somatic variant calling pipeline could reach 97% sensitivity and 100% precision respectively with near 48 million reads. The system is robust enough that when the number of reads was reduced to half of the original, the same sensitivity and precision in variant detection was reached (Table 2).

Taking advantage of the Cancer Cell Line Encyclopedia (CCLE) project that has characterized the commonly used human cancer cell lines with whole genome sequencing and/or whole exome sequencing, ActSeq was run with DNA samples extracted from 7 cell lines from our research lab (Table 3). Out of the 42 variants that are within the targeted region of ActSeq, 38 (90%) of them were detected by the ActSeq workflow. The 4 missed variants could be attributed to the lab-specific propagation of those cell lines, not a technical failure of the variant caller or the workflow, because upon manual review of the BAM files, all the 4 positions were covered with proper number of reads, the least for them is on *SF3B1* gene p.Q534 in HCC38 cells, but still at 462x (last column in Table 3). The allele frequencies of the 4 variants from CCLE data are 0.21~0.23, indicating they are not the dominant clone in the samples they were identified.

**Table 3:** CCLE cell lines variants as detected by ActSeq panel.

Cell_line	Gene	Entrez_ID	Chr	Start	End	Variant_Type	Ref	Alt	Protein.Change	CCLE_VAF	ActSeq_Pos	ActSeq_VAF	AD/DP
OVCAR5	*KRAS*	3845	12	25398284	25398284	Missense	C	A	p.G12V	0.98	Yes	1.00	1663/1666
OVCAR5	*PTPRD*	5789	9	8465638	8465638	Missense	C	A	p.R1181L	0.23	No	0.00	0/1763
OVCAR5	*RET*	5979	10	43596069	43596069	Missense	G	A	p.R79Q	0.43	Yes	0.50	134/268
OVCAR5	*CREBBP*	1387	16	3817760	3817760	Missense	C	T	p.A1071T	0.63	Yes	0.68	1223/1800
OVCAR8	*ERBB2*	2064	17	37880998	37880998	Missense	G	T	p.G776V	0.46	Yes	0.48	83/174
OVCAR8	*MSH6*	2956	2	48027301	48027301	Missense	A	T	p.T727S	0.46	Yes	0.50	454/915
OVCAR8	*CTNNB1*	1499	3	41266080	41266080	Missense	A	G	p.Q26R	0.36	Yes	0.34	534/1560
OVCAR8	*APC*	324	5	112174964	112174964	Missense	G	T	p.A1225S	0.20	Yes	0.37	631/1700
OVCAR8	*NOTCH1*	4851	9	139399891	139399891	Missense	G	A	p.P1486L	0.24	Yes	0.67	623/936
OVCAR8	*ATM*	472	11	108123578	108123578	Missense	G	T	p.V613L	0.45	Yes	0.53	921/1744
OVCAR8	*KRAS*	3845	12	25378636	25378636	Missense	G	T	p.P121H	0.39	Yes	0.47	555/1182
OVCAR8	*CREBBP*	1387	16	3820773	3820773	Nonsense	G	T	p.S893*	1.00	Yes	1.00	1297/1300
MDAMB231	*KRAS*	3845	12	25398281	25398281	Missense	C	T	p.G13D	0.53	Yes	0.66	642/972
MDAMB231	*TP53*	7157	17	7577099	7577099	Missense	C	T	p.R280K	0.96	Yes	1.00	1237/1243
MDAMB231	*BRAF*	673	7	140481417	140481417	Missense	C	A	p.G464V	0.52	Yes	0.48	549/1145
MDAMB231	*EPHA3*	2042	3	89499345	89499345	Nonsense	G	T	p.E839*	0.24	Yes	0.33	183/562
MDAMB231	*PDGFRA*	5156	4	55129981	55129981	Missense	A	T	p.Y172F	0.33	Yes	0.33	271/817
MDAMB231	*MSH3*	4437	5	80109433	80109433	Missense	G	A	p.G896R	0.49	Yes	0.34	327/966
MDAMB231	*NF1*	4763	17	29541474	29541475	Frame_Shift	-	C	p.T467fs	0.87	Yes	0.96	766/796
MDAMB468	*TP53*	7157	17	7577120	7577120	Missense	C	T	p.R273H	1.00	Yes	1.00	680/682
MDAMB468	*BRCA2*	675	13	32911387	32911387	Missense	G	C	p.M965I	0.23	No	0.00	0/1304
MDAMB468	*FANCA*	2175	16	89831471	89831471	Nonsense	G	A	p.Q869*	0.52	Yes	0.47	108/232
MDAMB468	*BCOR*	54880	X	39923194	39923194	Missense	C	G	p.E1172Q	0.98	Yes	0.99	304/306
MDAMB468	*ERBB2*	2064	17	37865585	37865585	Frame_Shift	G	-	p.G152fs	0.90	Yes	0.93	201/215
HCC38	*TP53*	7157	17	7577120	7577120	Missense	C	A	p.R273L	1.00	Yes	1.00	599/602
HCC38	*SF3B1*	23451	2	198268427	198268427	Missense	T	G	p.Q534P	0.21	No	0.00	0/462
HCC38	*PIK3CA*	5290	3	178927394	178927394	Missense	G	T	p.W386L	0.23	No	0.00	4/1763
HCC38	*TET2*	54790	4	106196816	106196816	Missense	C	A	p.H1717N	0.47	Yes	0.34	360/1062
HCC38	*PDGFRB*	5159	5	149502636	149502636	Missense	T	A	p.N718Y	1.00	Yes	1.00	704/704
HCC1806	*TP53*	7157	17	7577514	7577515	Frame_Shift	-	TT	p.T256fs	0.80	Yes	0.93	1098/1183
HCC1806	*PDGFRB*	5159	5	149499081	149499081	Missense	G	A	p.A916V	0.96	Yes	1.00	219/219
SKOV3	*PIK3CA*	5290	3	178952085	178952085	Missense	A	G	p.H1047R	0.52	Yes	0.48	1090/2257
SKOV3	*FBXW7*	55294	4	153247288	153247288	Missense	C	A	p.R505L	0.50	Yes	0.47	904/1907
SKOV3	*TP53*	7157	17	7579420	7579420	Frame_Shift	G	-	p.P89fs	0.86	Yes	0.90	358/399
SKOV3	*ARID1A*	8289	1	27058048	27058048	Nonsense	C	T	p.Q586*	0.50	Yes	0.49	121/247
SKOV3	*ROS1*	6098	6	117725466	117725466	Missense	G	C	p.L139V	0.43	Yes	0.44	299/686
SKOV3	*NRG1*	3084	8	32453468	32453468	Missense	A	G	p.N75D	0.19	Yes	0.03	28/965
SKOV3	*FLT3*	2322	13	28636197	28636197	Missense	A	C	p.S59A	0.56	Yes	0.62	397/643
SKOV3	*NF1*	4763	17	29653106	29653106	Missense	G	T	p.G1702C	0.45	Yes	0.50	841/1678
SKOV3	*CIC*	23152	19	42778253	42778253	Missense	T	C	p.F773S	0.48	Yes	0.45	233/517
SKOV3	*NCOA3*	8202	20	46262371	46262371	Missense	T	G	p.Y319D	0.52	Yes	0.50	866/1728
SKOV3	*APC*	324	5	112175952	112175952	Frame_Shift	A	-	p.E1554fs	0.16	Yes	0.03	57/1750

Note: AD, number of variant supporting reads in ActSeq; DP, total depth of reads in ActSeq.

### Concordance with orthogonal approach in variant detection on clinical specimens

**Fig 3:**
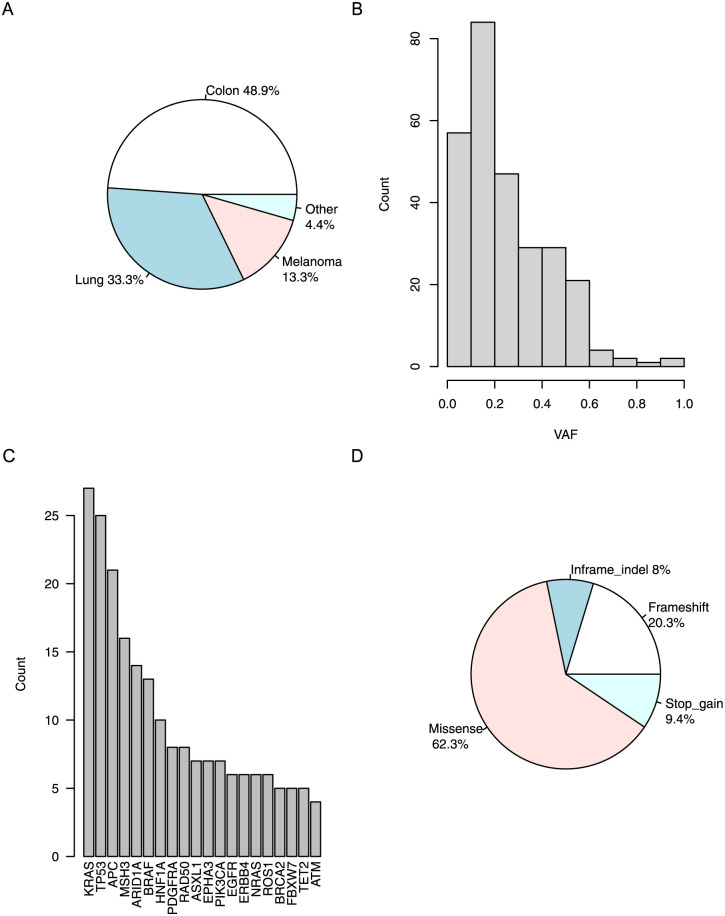
Clinical samples used in analytical validation. A) Source of clinical specimens, including 15 non-small cell lung cancer (Lung), 22 colorectal carcinoma (Colon), 6 melanoma, one gastric intestinal stromal tumor and one ovarian endometrioid carcinoma (Other). B) Distribution of the variant allele frequency of valid non-synonymous calls. C) Commonly mutated genes (top 20) in the cohort ranked by number of mutations as detected by ActSeq panel. D) Types of mutations in the cohort.

The MassARRAY-based OncoFOCUS panel (v3) was previously adopted in our community pathology lab as a fully validated clinical test of common mutations in *BRAF*, *EGFR*, *KIT*, *KRAS*, and *NRAS* genes, which are implicated in various cancers, particularly colon cancer, lung cancer and melanoma. Samples from a cohort of 45 formalin-fixed paraffin-embedded materials with known positive results from OncoFOCUS were tested with the ActSeq NGS panel for technical validation. The composition of cancer types is illustrated in Fig 3A. All the known variants detected by OncoFOCUS were discovered by the ActSeq NGS panel, and with comparable variant allele frequency (VAF) as listed in Table 4. Distribution of VAF of all valid non-synonymous variants detected by ActSeq panel is plotted in Fig 3B. More details on the variants are listed in S3 Table.

**Table 4:** Concordance of OncoFocus panel vs ActSeq panel.

					OncoFocus		ActSeq		
Patient	Gender	Age	Source	Gene	Mutation	Mut Freq	Mutation AA	Mutation CDS	VAF
H21001	Male	86	colon	*KRAS*	Q61H	0.30	p.Q61H	c.183A>T	0.25
H21002	Male	79	lung	*KRAS*	G12C	0.61	p.G12C	c.34G>T	0.59
H21003	Female	81	colon	*BRAF*	V600E	0.28	p.V600E	c.1799T>A	0.32
H21004	Male	86	melanoma	*BRAF*	V600K	0.19	p.V600K	c.1798_1799GT>AA	0.21
H21005	Female	64	Ovarian	*KRAS*	G13D	0.28	p.G13D	c.38G>A	0.15
H21006	Male	76	melanoma	*NRAS*	Q61L	0.16	p.Q61L	c.182A>T	0.16
H21007	Male	61	lung	*EGFR*	G719A	0.45	p.G719A	c.2156G>C	0.36
H21008	Male	84	colon	*KRAS*	Q61H	0.38	p.Q61H	c.183A>T	0.36
H21009	Male	76	colon	*KRAS*	G12V	0.52	p.G12V	c.35G>T	0.44
H21010	Female	90	melanoma	*NRAS*	Q61R	0.32	p.Q61R	c.182A>G	0.21
H21012	Female	63	colon	*BRAF*	V600E	0.19	p.V600E	c.1799T>A	0.24
H21013	Female	74	melanoma	*BRAF*	V600K	0.48	p.V600K	c.1798_1799GT>AA	0.51
H21014	Male	89	colon	*KRAS*	G12D	0.38	p.G12D	c.35G>A	0.38
H21015	Female	70	colon	*KRAS*	Q61H	0.39	p.Q61H	c.183A>C	0.38
H21016	Male	54	melanoma	*BRAF*	V600E	0.35	p.V600E	c.1799T>A	0.58
H21019	Female	68	melanoma	*BRAF*	V600K	0.49	p.V600K	c.1798_1799GT>AA	0.59
H21020	Male	59	colon	*KRAS*	G13D	0.22	p.G13D	c.38G>A	0.17
H21021	Male	56	colon	*KRAS*	G12V	0.44	p.G12V	c.35G>T	0.44
H21022	Female	77	lung	*EGFR*	L858R	0.48	p.L858R	c.2573T>G	0.64
H21023	Male	62	GIST	*KIT*	V559D	0.39	p.V559D	c.1676T>A	0.48
H21024	Male	79	colon	*KRAS*	G12A	0.41	p.G12A	c.35G>C	0.43
H21025	Male	69	lung	*KRAS*	G13D	0.45	p.G13D	c.38G>A	0.37
H21026	Male	64	colon	*KRAS*	A146T	0.52	p.A146T	c.436G>A	0.42
H21027	Male	82	colon	*BRAF*	V600E	0.25	p.V600E	c.1799T>A	0.24
H21029	Female	59	lung	*KRAS*	G12C	0.22	p.G12C	c.34G>T	0.19
H21030	Female	63	lung	*EGFR*	E746_A750del	0.81	p.E746_A750delELREA	c.2235_2249del15	0.74
H21031	Female	79	lung	*KRAS*	Q61H	0.44	p.Q61H	c.183A>C	0.40
H21032	Female	65	colon	*KRAS*	A146T	0.39	p.A146T	c.436G>A	0.31
H21033	Female	71	lung	*EGFR*	L858R	0.27	p.L858R	c.2573T>G	0.36
H21034	Female	77	lung	*KRAS*	G12S	0.22	p.G12S	c.34G>A	0.23
H21035	Female	53	lung	*EGFR*	L861Q	0.39	p.L681Q	c.2582T>A	0.40
H21036	Male	66	lung	*KRAS*	G12C	0.30	p.G12C	c.34G>T	0.27
H21037	Male	62	colon	*KRAS*	G12A	0.30	p.G12A	c.35G>C	0.33
H21038	Female	80	colon	*KRAS*	Q61R	0.18	p.Q61R	c.182A>G	0.25
H21039	Female	80	colon	*BRAF*	V600E	0.17	p.V600E	c.1799T>A	0.21
H21040	Male	68	colon	*KRAS*	G12D	0.23	p.G12D	c.35G>A	0.28
H21041	Female	67	lung	*KRAS*	G12C	0.59	p.G12C	c.34G>T	0.62
H21042	Female	62	lung	*BRAF*	G469A	0.36	p.G469A	c.1406G>C	0.40
H21043	Male	66	colon	*NRAS*	Q61K	0.45	p.Q61K	c.181C>A	0.46
H21044	Male	66	lung	*NRAS*	G12C	0.34	p.G12C	c.34G>T	0.33
H21045	Female	73	colon	*BRAF*	V600E	0.40	p.V600E	c.1799T>A	0.44
H21046	Male	82	colon	*KRAS*	G12D	0.38	p.G12D	c.35G>A	0.46
H21047	Female	74	colon	*BRAF*	V600E	0.25	p.V600E	c.1799T>A	0.30
H21048	Female	63	lung	*KRAS*	G12D	0.16	p.G12D	c.35G>A	0.15
H21049	Male	69	colon	*NRAS*	Q61H	0.27	p.Q61H	c.183A>T	0.18

Higher yield in variant detection from NGS panel sequencing is obvious as in Fig 3C, which illustrates the top 20 genes with SNV/indel variants in the 45 patient specimens. *KRAS* and *TP53* are unsurprisingly the top 2 genes with the highest number of variants in this cohort. With the OncoFOCUS panel, only 1 variant was found from each patient. With the ActSeq NGS panel, a total of 276 variants from 67 genes were discovered, at least 2 variants per patient (range of 2–19 variants per patient). The variant types are illustrated in Fig 3D, 62% of them are missense mutations, 28% are indels (in-frame or frameshift) and 9% are stop-gains.

## Discussion

The completion of The Cancer Genome Atlas (TCGA) project consolidated our understanding of the major oncogenic signaling pathways, providing a more complete picture of drivers in oncogenesis [16]. The National Cancer Institute Molecular Analysis for Therapy Choice (NCI-MATCH) trial demonstrated the feasibility and efficacy of using NGS to triage patients to investigational therapy [17]. The use of NGS panels in general clinical oncology practice is rapidly increasing as well. Often, the limited amount of available tissue encourages a high-throughput approach, particularly in non-small cell lung cancer, wherein the availability of specimens after initial pathological diagnosis is often restricted. A targeted clinical NGS assay for high-throughput tumor profiling would thus meet the increasing need in oncology practice. While commercially established NGS panels for clinical oncology utility are available, it remains advantageous to develop a customized NGS panel in-house within a molecular diagnostic laboratory. Potential advantages may include faster turn-around-time, better use of local resources and improved communication with clinicians. Our efforts in developing and evaluating the ActSeq panel reflect these parameters.

ActSeq is a customized NGS panel that aims to cover clinically actionable genes and is an intended upgrade from the mass spectrometry-based OncoFOCUS panel that was previously adopted. It is an expansion from 5 genes with specified variants to all exons of 141 genes. The choice of sequencing strategy for a clinical test has important ramifications on the variant calling process, as clearly there are advantages of hybridization over amplicon based approaches in terms of target enrichment [18]. On the other hand, however, hybridization capture methodology is prone to off-target enrichment. With MYBAIT used in the ActSeq panel, the short length of probes makes it more vulnerable, particularly when the probes bear similarity with non-coding sequences. Indeed, a high percentage off-bait reads, which ranged from 53%~58% in the first three runs, was observed. However with sufficient coverage of the targeted regions (727x~879x), this does not negatively affect downstream variant calling. It is also possible to reduce off-bait reads via improving wet-lab procedures in future iterations of this assay.

Depth and breadth of sequencing coverage on targeted regions are critical factors in NGS panel performance and have a deep impact on the variant calling. A typical average read depth in a NGS panel assay is 100~500x [19]. The ActSeq panel has a mean target coverage over 700x. When tested with NA12878, our panel obtained high sensitivity (98%) and high PPV (98%), which not only verified the success of the bioinformatics workflow for variant calling but also indicated the proper coverage in the targeted regions. The higher depth and high percentage of targeted region achieved in this panel ensures that it is possible to detect somatic variants at low allele frequency. This is particularly important, since in cancer tissue the presence of non-cancerous components is inevitable. In a real-world scenario, tissues with low tumor purity or sub-clonal events due to tumor heterogeneity would be encountered, a significant portion of clinically actionable variants have low allele frequencies [20]. Therefore, higher coverage is always preferred whenever possible.

Clinical tumor specimens are often in the form of FFPE materials, wherein DNA degradation and modification may impact library preparation and variant detection. However it has already been found that with higher coverage, reliable mutation calling is possible [21,22]. In current practice, most NGS panels in clinical oncology use FFPE materials. We tested the performance of FFPE vs fresh-frozen tissues in library preparation and sequencing and no difference was found. In addition, to meet the reality of the wide variation in the amount and quality of DNA from clinical specimens, we have established a protocol that can handle as little as 13 ng starting DNA, enabling analysis of lower quality FFPE materials.

Assessing the performance of a bioinformatic workflow, particularly the metrics of variant calling for NGS assays is critical and challenging since ground truth for clinical samples is rarely known and variants cannot be individually validated or quality controlled. However, in recent years standard reference materials with a well-curated variant truth set, including NA12878 cell DNA (Genome-in-a-bottle, for germline use) and OncoSpan HD827 DNA (from Horizon Discovery, for somatic use) used in the current report, have been developed by NIST [15] and commercial entities. These efforts have greatly facilitated the benchmarking of NGS panel development. We obtained high sensitivity and precision in both germline and somatic variant calling. By down sampling, we found that reducing the reads to half the amount does not affect the performance of our Mutect2 based variant calling pipeline, as we detected the same number of variants in the OncoSpan HD827 sample corresponding to our target regions (134 variants) as with the full set of reads. When compared to 45 clinical FFPE samples that were previously tested with an OncoFOCUS panel, a mass spectrometry-based mutational detection approach, we have confirmed that all the known mutations were identified by the ActSeq panel test, and in comparison, an apparent advantage with the NGS panel is higher diagnostic yield.

There are also obvious limitations of the ActSeq panel, including the lack of capability to determine copy number variation (CNV) and structure variation (SV) detection, as these events are commonly involved in oncogenesis. Amplification of certain oncogenes, such *ERBB2* and *EGFR*, are clinically relevant to targeted therapeutics. Many SV events are of diagnostic value, especially those in leukemia and soft tissue sarcoma. Microsatellite instability index (MSI) and tumor mutational burden (TMB) are also not addressed in this panel. All of these features, plus additional genes or variants, could be added in future continuous development of the panel. The aforementioned high percentage of off-bait reads of the panel in this current form means there is room for improvement in cost reduction. However, since many different cancer genes could be included in a given panel, NGS allows consolidation of the clinical laboratory workflow, leading to further efficiency and savings. In the long run, the wide adoption of NGS in healthcare would be beneficial and cost-effective [23].

It should be noted that while the NGS related bioinformatics tools and workflow construction have matured over recent years, variant filtering, annotation, prioritization and clinical interpretation remain to be daunting tasks for clinical labs newly starting NGS assays. To extract those variants with tangible clinical value and biological significance to a meaningful report ready for sign-out requires more dedicated tools and resources customized to the actual needs of the clinical lab.

In summary, we developed a NGS panel with a focus on clinically actionable mutations and validated the performance in library construction, sequencing and variant calling. Full concordance with an orthogonal mutation detection approach was observed in 45 clinical specimens. Our results illustrate the feasibility for a panel development in a community pathology lab, suitable for clinical application, improving diagnosis, prognosis and personalized therapeutic decisions.

## Supporting information

S1 Fig Genes and baits distribution on the genome. (EPS)

S2 Fig ActSeq NGS panel bioinformatics workflow. (EPS)

S1 Table Gene list of ActSeq panel. (CSV)

S2 Table Bait (probe) sequences. (FASTA)

S3 Table Variants detected in clinical samples. (TXT)
